# Comparing IM Lorazepam and IM Clothiapine for Agitated Psychosis in Hospitalized Patients

**DOI:** 10.1192/j.eurpsy.2024.686

**Published:** 2024-08-27

**Authors:** •. Kverashvili, E. Malik, •. Shelef, •. Stryjer

**Affiliations:** ^1^Psychiatry, Abarbanel Mental Health Center, Bat-Yam; ^2^Psychiatry, Faculty of Medicine, Tel-Aviv University, Tel-Aviv; ^3^Psychiatry, Lev Hasharon Mental Health Center, Netanya, Israel

## Abstract

**Introduction:**

When patients in a closed hospital ward experience acute psychosis and become highly agitated or pose a risk to themselves and others, it’s often crucial to provide immediate sedative treatment. However, there is currently no consensus on whether the preferred medication for these situations should be antipsychotic drugs or benzodiazepines.

**Objectives:**

This study aimed to compare how well a single intramuscular dose of 2-4 mg Lorazepam performs against 40 mg Clothiapine in terms of effectiveness and side effects. These treatments were administered as immediate emergency measures to patients experiencing psychosis with severe agitation or behaviors that posed a risk to themselves or their surroundings.

**Methods:**

We conducted a retrospective clinical study involving 100 patients experiencing aggressive psychosis. These patients were divided into two groups. The first group comprised 50 patients who received a single intramuscular (IM) dose of up to 40 mg Clothiapine. The second group consisted of 50 patients who received IM treatment with 2-4 mg Lorazepam. We assessed the patients’ outcomes around 8 hours after treatment or upon receiving any additional treatment.

**Results:**

There were no significant statistical differences in the demographic and clinical characteristics (e.g., age, gender, number of hospitalizations, duration of illnesses, psychiatric diagnosis, comorbidity) of the patients between the two groups (p > 0.05).

Before treatment, there were no statistical differences in the severity of clinical symptoms (CGI-S) between the two groups [CGI-S (Mean ± SD): 5.32 ± 1.09 vs. 5.38 ± 1.4, p = 0.8].

However, in the Clothiapine group, a statistically significant clinical improvement (CGI-I) was observed after treatment [CGI-I (Mean ± SD): 2.42 ± 0.9 vs. 1.96 ± 1.16, p = 0.029 *].

There were no significant differences in the need for physical restraint or additional medication following the initial treatment between the two groups (p > 0.05).

Furthermore, there were no statistically significant differences in the major side effects of the drugs, the necessity for referral to the general emergency room, or incidents of falls (p > 0.05).

**Conclusions:**

When dealing with a psychotic state marked by severe agitation or threats to oneself and others, the use of IM Clothiapine as a treatment option may offer certain advantages over IM Lorazepam. Importantly, these advantages come without significant exposure to side effects or potential risks associated with Clothiapine.

**Disclosure of Interest:**

None Declared

